# RNA-Seq analysis of differentially expressed genes of *Staphylococcus epidermidis* isolated from postoperative endophthalmitis and the healthy conjunctiva

**DOI:** 10.1038/s41598-020-71050-6

**Published:** 2020-08-28

**Authors:** Qing Liu, Nan Chen, Huabo Chen, Yusen Huang

**Affiliations:** 1 Qingdao Eye Hospital of Shandong First Medical University, Qingdao, China; 2 State Key Laboratory Cultivation Base, Shandong Provincial Key Laboratory of Ophthalmology, Shandong Eye Institute, Shandong First Medical University and Shandong Academy of Medical Sciences, 5 Yanerdao Road, Qingdao, 266071 China

**Keywords:** Microbiology, Diseases

## Abstract

*Staphylococcus epidermidis* (*S. epidermidis*) is one of the primary pathogens in postoperative endophthalmitis, which is a devastating complication of cataract surgery and often results in irreversible visual loss and even blindness. Meanwhile, it is the most frequently isolated commensal bacterium in the healthy conjunctiva. In this study, we investigated the differentially expressed genes (DEGs) of *S. epidermidis* isolated from the patients with postoperative endophthalmitis and the healthy conjunctiva to predict their functions and pathways by Illumina high-throughput RNA sequencing. Using genome-wide transcriptional analysis, 281 genes (142 upregulated and 139 downregulated genes) were found to be differentially expressed (fold change ≥ 2, *p* ≤ 0.05) in the strains from endophthalmitis. Ten randomly selected DEGs were further validated by quantitative reverse transcription polymerase chain reaction (qRT-PCR). GO enrichment analysis suggested that more DEGs were associated with the thioredoxin system and iron ion metabolism. KEGG pathway analysis revealed that more DEGs were associated with the pathways of the two-component system and pyruvate metabolism. Moreover, the gene SE1634 code for staphylococcal toxin was significantly upregulated in *S. epidermidis* strains of the endophthalmitis, which might be directly responsible for the pathogenesis of endophthalmitis. In conclusion, this research is helpful for further investigations on genes or pathways related with the pathogenesis and therapeutic targets of *S. epidermidis* endophthalmitis.

## Introduction

Postoperative endophthalmitis is a rare but devastating complication of cataract surgery, often resulting in irreversible visual loss and even blindness if it is not treated properly and in time. Despite effective intervention strategies during ophthalmic surgery, the incidence of postoperative endophthalmitis ranged from 0.033% to 0.36% over the past decade^1–3^.

Monitoring the etiologic organisms of endophthalmitis through culture of the vitreous or aqueous humor and their antibiotic resistance is critical for the empiric management of endophthalmitis. Coagulase-negative staphylococcus, particularly *Staphylococcus epidermidis* (*S. epidermidis*), has been reported as the most frequently isolated bacterial species in endophthalmitis^4–6^. Bacteria can enter the anterior chamber at any time during intraocular surgery at a rate of 20–40%, with *S. epidermidis* being the most commonly cultured organism^7–9^. Furthermore, there is evidence that the microbial flora colonizing the normal conjunctiva is responsible for postoperative endophthalmitis^7,10,11^. *S. epidermidis* seems to be predominantly present as normal flora in skin and mucous membranes, as well as the healthy conjunctiva^12–14^. Generally, *S. epidermidis* does not produce many toxins and tissue-damaging exoenzymes as *Staphylococcus aureus* does*.* However, *S. epidermidis* can develop from commensals to opportunistic pathogens depending on the adhesion factors, evasion of the host’s immune system, and production of virulence factors^15^. The δ-toxin is a main hemolytic toxin produced by *S. epidermidis* and may cause the lysis of erythrocytes and proinflammation^16^. Besides, recent research has identified phenol-soluble modulins (PSMs) in *S. epidermidis*, which have a great contribution to infection^16,17^. Therefore, it is essential to assess genetic differences of *S. epidermidis* isolated from postoperative endophthalmitis and the normal conjunctiva.

Previous studies on the comparison of *S. epidermidis* isolates obtained from endophthalmitis and the normal conjunctiva are often limited to the differences in phenotypic and genetic characterization^18–20^. The advent of the next generation sequencing allows the whole-genome, transcriptome, and even epigenomics sequencing of organisms. A full understanding of the transcriptome helps to interpret the functional elements of the genome and hypothesize the potential mechanisms of physiological and pathological conditions^21^. High-throughput RNA sequencing (RNA-Seq) has been performed for transcriptome analysis as an alternative to other transcriptomic technologies such as microarrays because of its advantages of covering a large dynamic range, possessing a high level of reproducibility, and requiring fewer RNA samples^21^. This tool is often used to identify differences in gene expression between biological samples^22^. Abundant results related to prokaryotic organisms such as *E. coli*, *Salmonella typhi*, and *Helicobacter pylori* have been achieved^23–25^.

In this study, we aimed to investigate the differentially expressed genes (DEGs) among the *S. epidermidis* isolates obtained from the eyes with postoperative endophthalmitis versus those from the conjunctival sac of healthy individuals by Illumina high-throughput RNA-Seq technology. We also attempted to perform Gene Ontology (GO) and Kyoto Encyclopedia of Genes and Genomes (KEGG) pathway enrichment analyses of the DEGs and associated pathway genes involved in the pathogenesis and potential therapeutic targets of *S. epidermidis* related endophthalmitis.

## Materials and methods

### Preparation of *Staphylococcus epidermidis*

**Figure 1:**
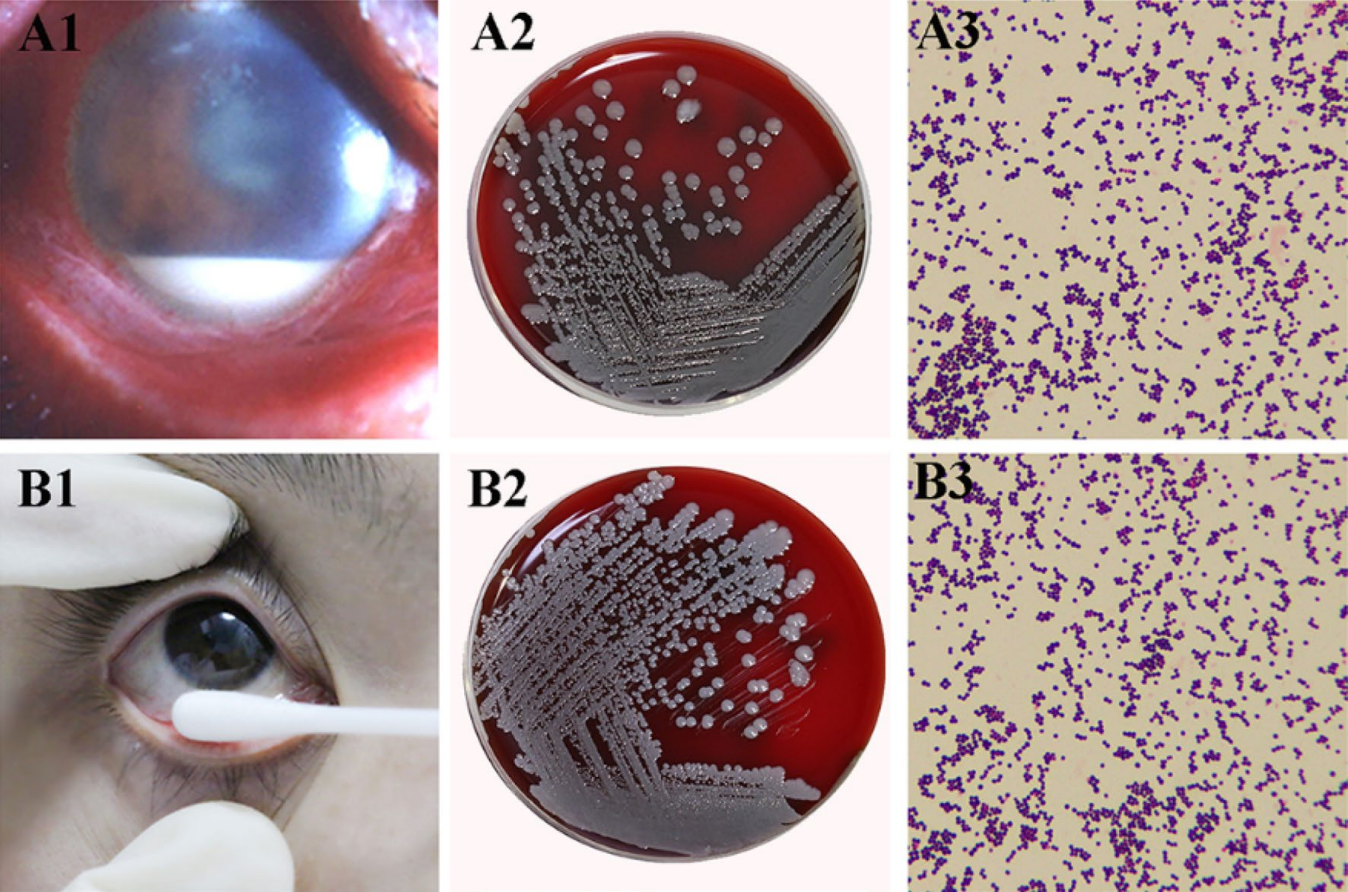
Preparation of *S. epidermidis.* (**A1**) A slit lamp photo of a patient with postoperative endophthalmitis. (**A2**) The positive sample of the vitreous after culture for 24 h on a blood agar plate identified as *S. epidermidis* by the automated microbiology system. (**A3**) Gram staining (× 1,000). (**B1**) A photo of the conjunctival swab for a healthy individual. (**B2**) The positive sample after culture for 24 h on blood agar plate identified as *S. epidermidis* by the automated microbiology system. (**B3**) Gram staining (× 1,000).

Five strains of *S. epidermidis* (P-Se) were isolated from the vitreous of five eyes (five patients) with post-cataract surgery endophthalmitis in the Clinical Laboratory of the Qingdao Eye Hospital of Shandong First Medical University. All patients had ocular pain, decreased vision, anterior and posterior segment inflammation, and hypopyon, as well as culture-proven (vitreous) endophthalmitis caused by *S. epidermidis* (Fig. 1A1–A3 and Table 1). Meanwhile, five strains of commensal *S. epidermidis* (NP-Se) were isolated from the conjunctival sac of five healthy individuals using swabs (Fig. 1B1–B3).

**Table 1:** Clinical characteristics of all five patients with post-cataract endophthalmitis.

Patient no.	Age (years)	Sex	Diagnosis	Onset time (days)	Vitreous isolates
1	64	Male	Post-cataract endophthalmitis	6	*S. epidermidis*
2	64	Male	Post-cataract endophthalmitis	12	*S. epidermidis*
3	58	Female	Post-cataract endophthalmitis	11	*S. epidermidis*
4	68	Male	Post-cataract endophthalmitis	2	*S. epidermidis*
5	61	Female	Post-cataract endophthalmitis	8	*S. epidermidis*

All clinical samples were inoculated onto blood agar plates (Auto Biotechnology, Zhengzhou, China) and incubated at 37 °C with 5% carbon dioxide for 24 h (Thermo, Massachusetts, USA). *S. epidermidis* isolates were considered when the incubation exhibited single colony morphology, Gram stain was positive, Catalase test was positive, and Oxidase test was negative. Then the ten pure strains of *S. epidermidis* were identified by an automatic microbiological identification and susceptibility analysis system (Beckman Coulter WalkAway-96 plus, California USA), which uses colorimetry and fluorescence to identify both gram-negative and gram-positive organisms through a variety of biochemical reactions.

The study was approved by the ethics committee of Shandong Eye Institute. Based on the tenets of the Declaration of Helsinki, written informed consents were obtained from every enrolled participant.

### RNA extraction

For the ten strains of *S. epidermidis*, the phenol water (Sinopharm Chemical Reagent Co., Shanghai, China) was added and shaken vigorously to pyrolyze each sample. All centrifugal tubes were incubated with an oscillating metal bath at 65 °C for a maximum speed shock for 30 to 60 min. After 5 min of cooling on ice, centrifugation was performed at 12,000×*g*, 4 °C for 10 min, and then the supernatant water was transferred into a new centrifugal tube. The purification of RNA was performed using the TRK-1002 (Lianchuan Bio, Hangzhou, China) and following the manufacturer's recommendation. The quality of each extracted RNA was checked using the Bioanalyzer 2100 (Agilent Technologies, Santa Clara, CA, USA) with an RNA integrity number (RIN) > 7.0 for cDNA library preparation, and the required RNA samples were stored in a refrigerator at − 80 °C before they were sent to Lianchuan Bio for RNA-Seq.

### cDNA library construction and sequencing

Five-μg RNA of each sample was used to build a cDNA library. Ribosomal RNA (rRNA) was depleted using the Ribo-Zero Magnetic Kit (Epicentre, Madison, WI, USA) before the library was generated using the Illumina Truseq RNA Sample Preparation Kit (Illumina, San Diego, CA, USA) according to the manufacturer’s protocol. On the basis of the cleaved RNA fragments, cDNA was synthesized using Uracil-N-Glycosylase (UNG), and a cDNA cluster was generated according to a cBot User Guide to PCR amplification after ligation of adaptors. Illumina sequencing was carried out on an Illumina HiSeq™ 2000 platform (Lianchuan Bio).

### RNA-Seq data analysis

After sequencing, image data was transformed into raw reads and stored in FASTQ format for per sample. The resulting clean reads were obtained by removing the low-quality, adapter, poly-N containing, and shorter-than-70 bp reads, and mapped to the reference genome of *S. epidermidis* strain ATCC12228 (https://www.ncbi.nlm.nih.gov/genome/155?Genomeassembly_id = 299299) using software Rockhopper^26^. Gene expression was normalized by calculating Reads per Kilobase per Million Mapped Reads (RPKM) as described by Mortazavi and colleagues^27^, which could eliminate the effects of gene length and the differences of sequencing quantity when calculating the gene expression. DEGs were determined using the edgeR package of R software^28^, and the false discovery rate ≤ 0.05 and the absolute value of log_2_ fold change ≥ 1 (|log_2_ FC|≥ 1) were used as the threshold to determine the statistically significant differences in gene expression^29^. To obtain as much information as possible, GO^30^ and KEGG^31–33^ pathway analyses were performed to identify significant functions of the DEGs. The Fisher’s exact test was used to determine the enrichment in categories.

### Quantitative reverse transcription polymerase chain reaction (qRT-PCR)

To verify the accuracy of the RNA-Seq data, qRT-PCR analysis of ten randomly selected DEGs (including five upregulated and five downregulated genes) was performed using the same extracted total RNA as the RNA-Seq analysis in each of the ten samples. Briefly, one microgram of RNA was used for reverse transcription with the TURE script 1st Stand cDNA SYNTHESIS Kit (Aidlab, Beijing, China) according to the manufacturer's instructions, and gene-specific primer pairs were designed with Beacon Designer 7 based on transcriptome-assembled data. The primer sequence pairs are shown in Table 2. PCR amplifications were carried out using the qTOWER Real-Time PCR Thermal Cycler (Analytik JenaAG, Jena, Germany). The reaction was proceeded under the following conditions: 3 min at 95 °C, 10 s at 95 °C, and 30 s at 58 °C before the plate reads were taken, 10 s at 95 °C, 39 cycles, and final Melt curve analysis (60–95 °C, + 1 °C/cycle, holding time 4 s). The relative quantification of gene expression was computed using the 2^−ΔΔCt^ (C_T_^reference gene^ − C_T_^target gene^) method with 16S rRNA as the reference gene^34^. All experiments were performed in triplicates to ensure accuracy.

**Table 2:** Primers of validated genes.

Name	Forward	Reverse
16sRNA	5′-TCCTACGGGAGGCAGCAGT-3′	5′-GGACTACCAGGGTATCTAATCCTGTT-3′
SE1763	5′-TCAATGGAGAGTAGCAGATA-3′	5′-TCCGAAAGTAGAACCAATAC-3′
SE0727	5′-AGAATTTTATGACGGTTTAAGAGG-3′	5′-ATCGCTTATGATTGATAACTGTCT-3′
SE1634	5′-GGCAGCAGATATCATTTCTAC-3′	5′-ATCCATTTTACTAAATCACCG-3′
SE0124	5′-AACGCCCGAATTATTTAATGTC-3′	5′-ACTTTGATGTGCTGTATAACCA-3′
SE0125	5′-GTGAGCGAAAAAGAGTTATTG-3′	5′- ACGTATACTTTGGTTCACCGT-3′
SE2185	5′-CAATGGGCAACAACAACAA-3′	5′-TTCGCTTCATCATACTCAGTC-3′
SE2389	5′-TCGGTCTAATCACACAAAGTT-3′	5′-AGAGGAAACATAATGGAGAAAGTC-3′
SE2184	5′-AGAGGTATTGAGGTTGACGATT-3′	5′-CAAGGATATGGGCAGCGATA-3′
SE0241	5′-CGGTTAGTGTGACGATTCT-3′	5′-CAGTTGCTTCTTGTGTTAGTT-3′
SE2183	5′-TGGTTCGCAAATTAAATGGA-3′	5′-TGGTTAGGATTATCACAAGGTA-3′

### Statistical analysis

Statistical analysis was performed using Prism 6 (GraphPad Software, La Jolla, CA, USA). Differences between groups were assessed using the Student's t-test or Mann–Whitney U test. Validated data from qRT-PCR are expressed as the mean ± standard deviation (SD). A *p* value < 0.05 was considered statistically significant.

## Results

### Reads generation

Ten cDNA libraries prepared from the 10 strains of *S. epidermidis* were sequenced on an Illumina HiSeqTM 2000 platform. A total of 234,631,384 raw reads were generated, and after the sequencing adapters and low-complexity reads were removed, 213,509,502 clean reads were generated with an average of 21,059,504 reads in the P-Se cDNA libraries and 21,642,396 reads in the NP-Se cDNA libraries. The clean reads which were perfectly mapped to the reference genomes without mismatch were all greater than 70%. A detailed summary of the sequencing results is shown in Table 3.

**Table 3:** The RNA-Seq data for 10 samples.

Sample ID	Clean reads	Total bases	Mapped reads (%)	Q20%	Q30%	GC%
NP-Se1	21,013,380	3,145,789,492	87.75	99.04	97.00	36.14
NP-Se2	22,165,878	3,311,787,836	77.27	98.87	96.66	35.18
NP-Se3	22,087,006	3,320,714,645	82.23	99.35	97.84	34.75
NP-Se4	25,116,706	3,756,674,477	84.75	99.00	96.99	34.27
NP-Se5	17,829,010	2,664,145,425	86.27	99.22	97.35	37.85
P-Se1	18,396,772	2,740,094,564	71.31	98.7	96.18	37.17
P-Se2	22,337,566	3,337,529,936	88.97	99.13	97.33	34.09
P-Se3	21,174,054	3,170,408,868	88.23	99.12	97.20	34.33
P-Se4	18,760,720	2,820,137,111	87.93	99.34	97.81	34.83
P-Se5	24,628,410	3,692,307,247	78.66	99.20	97.44	35.53

Q20% means the sequencing error rate of the base was less than 1%; Q30% means the sequencing error rate of the base was less than 0.1%.

### Differentially expressed gene analysis

**Figure 2:**
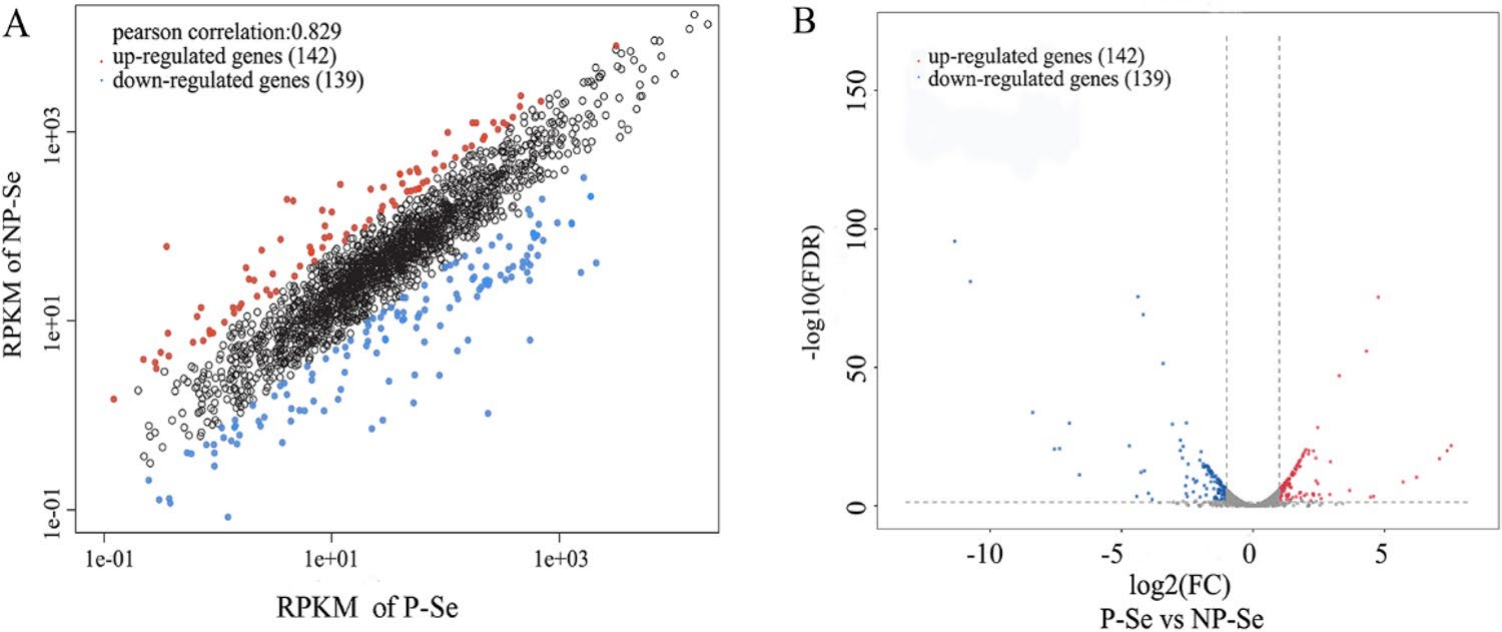
RNA-Seq analysis of differentially expressed genes (DEGs) of *S. epidermidis* isolates between postoperative endophthalmitis (P-Se) and the healthy conjunctiva (NP-Se). (**A**) A scatter plot demonstrating the expression variation of genes in two groups. The values of x- and y-axes represent the normalized signal values of the samples. (**B**) A volcano plot showing the DEGs in two groups with FDR ≤ 0.05 and |log2 FC|≥ 1 as the threshold. The red dots represent 142 significantly upregulated genes, and the blue dots represent 139 significantly downregulated genes in the group of P-Se compared with their expression levels in the group of NP-Se.

A total of 281 DEGs were identified between postoperative endophthalmitis isolates and healthy conjunctival isolates, of which 142 were upregulated and 139 were downregulated. A scatter plot was used to assess the expression variation of the genes between P-Se and NP-Se (Fig. 2A). Moreover, a volcano plot was constructed to visualize the DEGs between two groups (Fig. 2B). The annotation of the 281 DEGs in the NCBI-nr database shows that the gene codes for Staphylococcal toxin (SE1634) and phenol-soluble modulins β1 (SE0846) were significantly upregulated in P-Se, which might be directly responsible for pathogenesis of *S. epidermidis* endophthalmitis. A complete list of all DEGs is shown in Supplementary Table S1.

### Validation by qRT-PCR

**Figure 3:**
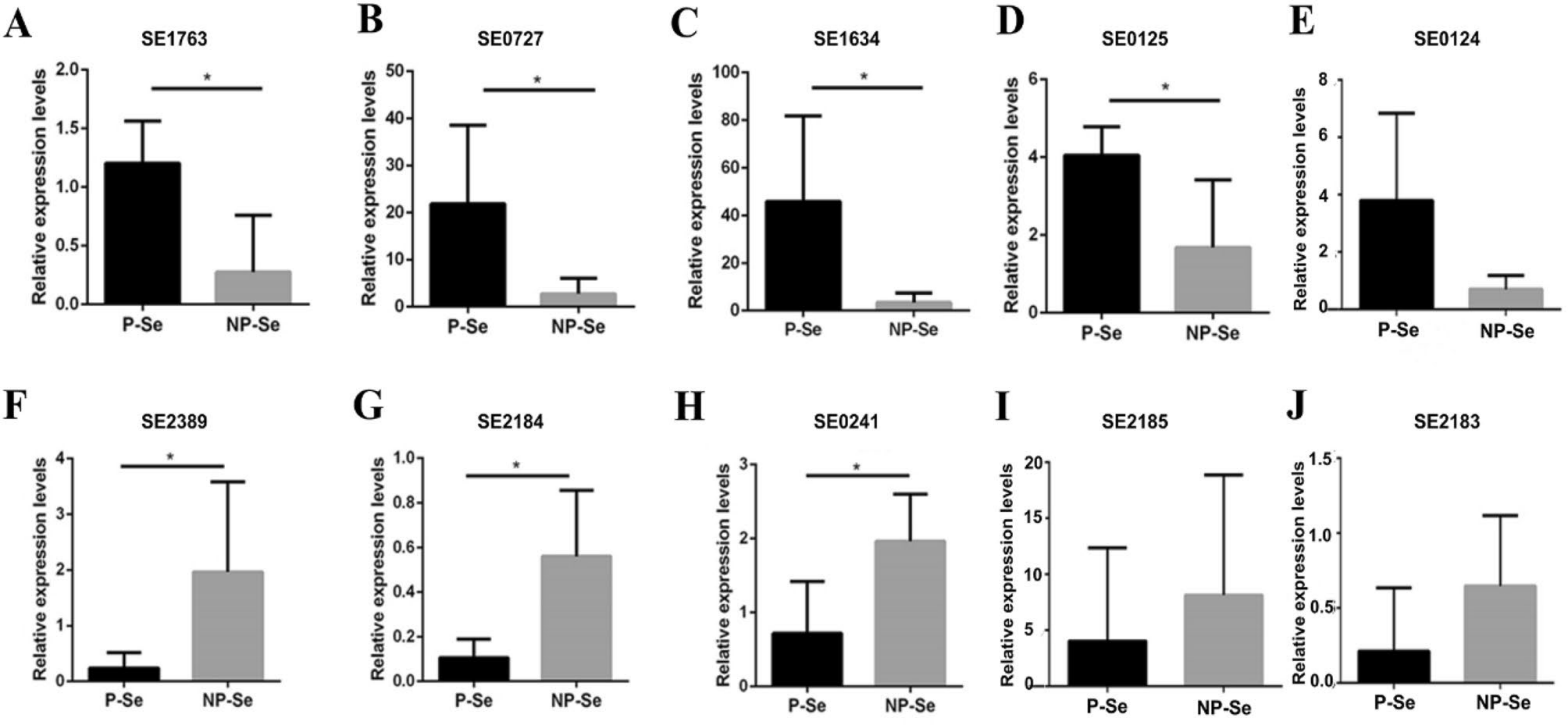
qRT-PCR analysis for validation of expression levels of ten randomly selected DEGs. SE1763 (**A**), SE0727 (**B**), SE1634 (**C**), and SE0125 (**D**) were significantly upregulated in the group of P-Se compared to NP-Se, while SE0124 (**E**) had no significant difference. Similarly, the genes SE2389 (**F**), SE2184 (**G**), and SE0241 (**H**) were significantly downregulated in the group of P-Se compared to NP-Se, while SE2185 (**I**) and SE2183 (**J**) were of no significant difference.*p < 0.05.

**Figure 4:**
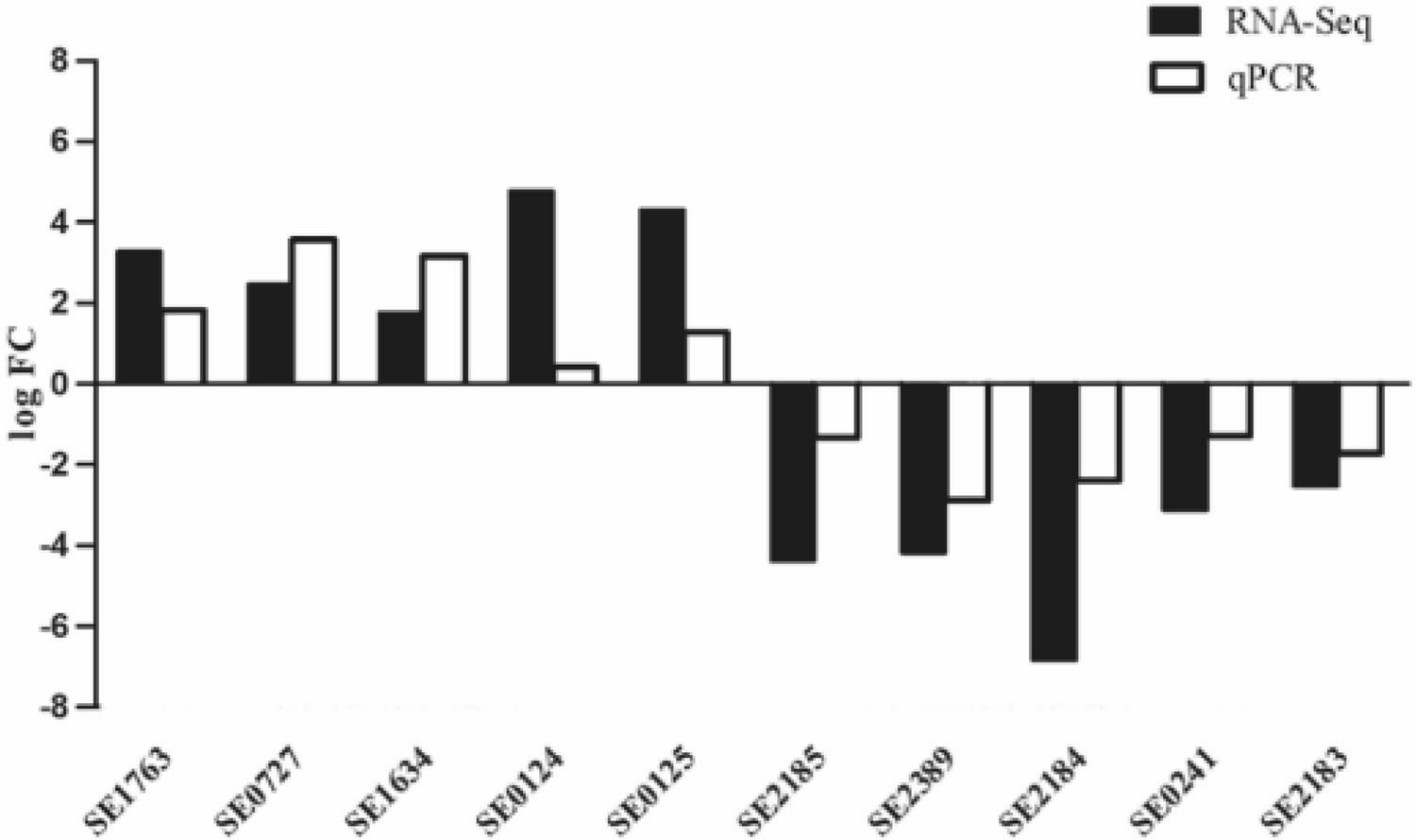
Comparison of the log2 fold change (log FC) of ten randomly selected differentially expressed genes between RNA-Seq and qRT-PCR. The results indicated that the qRT-PCR data were consistent with the results of RNA-Seq.

To validate the results of RNA-Seq, ten DEGs (including five upregulated and five downregulated genes) were randomly selected for qRT-PCR analysis on the basis of the fold-change and *p* values. The expression levels of SE1763, SE0727, SE1634, SE0124, and SE0125 were upregulated, while those of SE2185, SE2389, SE2184, SE0241, and SE2183 were downregulated. Specifically, the genes SE1763, SE0727, SE1634, and SE0125 were significantly upregulated in the group of P-Se compared to NP-Se (Fig. 3A–D), while SE0124 (Fig. 3E) had no significant difference. Similarly, the genes SE2389, SE2184, and SE0241 were significantly downregulated in the group of P-Se compared with their levels in the NP-Se group (Fig. 3F–H), while SE2185 (Fig. 3I) and SE2183 (Fig. 3J) were of no significant difference. In addition, we compared the log2 fold change of ten selected DEGs between RNA-Seq and qRT-PCR (Fig. 4). The qRT-PCR data were consistent with the RNA sequencing data, indicating the reliability of the RNA-Seq results.

### GO functional analysis of DEGs

**Figure 5:**
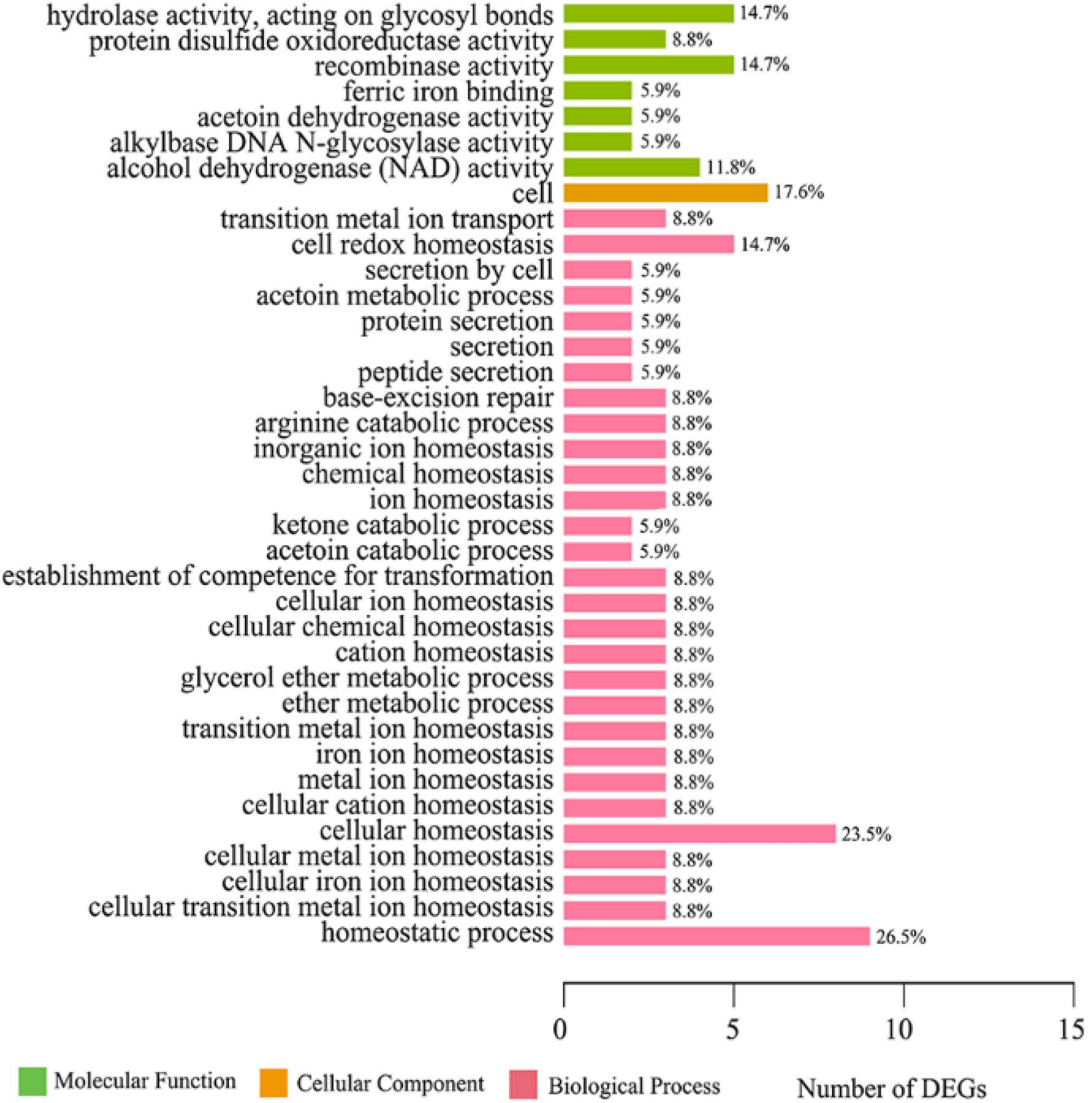
Gene Ontology (GO) enrichment analysis of 281 differentially expressed genes. The top three enriched terms in the category of biological process (BP) were homeostatic process, cellular homeostasis and cell redox homeostasis. In the category of cellular component (CC), cell was the only enriched term. The terms of hydrolase activity, acting on glycosyl bonds, recombinase activity, and alcohol dehydrogenase (NAD) activity were dominant in the molecular function category (MF).

GO annotation is an international standard system of gene function classification that has three main categories to describe biological process (BP), cellular component (CC), and molecular function (MF). To gain an insight into the biological roles of the most significantly up- or downregulated genes, GO enrichment analysis was performed using the Fisher’s exact test with *p* value ≤ 0.05 as the threshold. In brief, all DEGs were enriched to 37 GO terms and classified into categories of BP with 29 GO terms, CC with 1 GO term, and MF with 7 GO terms. As shown in Fig. 5 and Supplementary Table S2, the top three enriched terms in BP were “homeostatic process (GO: 0042592)”, “cellular homeostasis (GO: 0019725)”, and “cell redox homeostasis (GO: 0045454)”, with the number of DEGs being 9, 8, and 5, respectively. In the CC category, “cell (GO: 0005623)” was the only but highly enriched term, with 6 DEGs attached. In the category of MF, genes were significantly enriched with the terms of “recombinase activity (GO: 0000150)”, “hydrolase activity, acting on glycosyl bonds (GO: 0016798)”, and “alcohol dehydrogenase (NAD) activity (GO: 0004022)”, and the number of DEGs being 5, 5, and 4, respectively. Moreover, by analyzing these involved genes we found several DEGs were closely associated with the thioredoxin system, which plays a crucial role in defense against oxidative stress and provides an opportunity to kill bacteria^35^.

Several terms were also strongly associated with the BP of iron ions. The terms of “ferric iron binding (GO: 0008199)”, “cellular iron ion homeostasis (GO: 0006879)”, and “iron ion homeostasis (GO: 0055072)” were significantly enriched. Iron acquisition strategy is required for pathogenic bacterial colonization and subsequent pathogenesis^36^. More information about the involved genes of GO enrichment is shown in Supplementary Table S2.

### KEGG pathway analysis of DEGs

**Figure 6:**
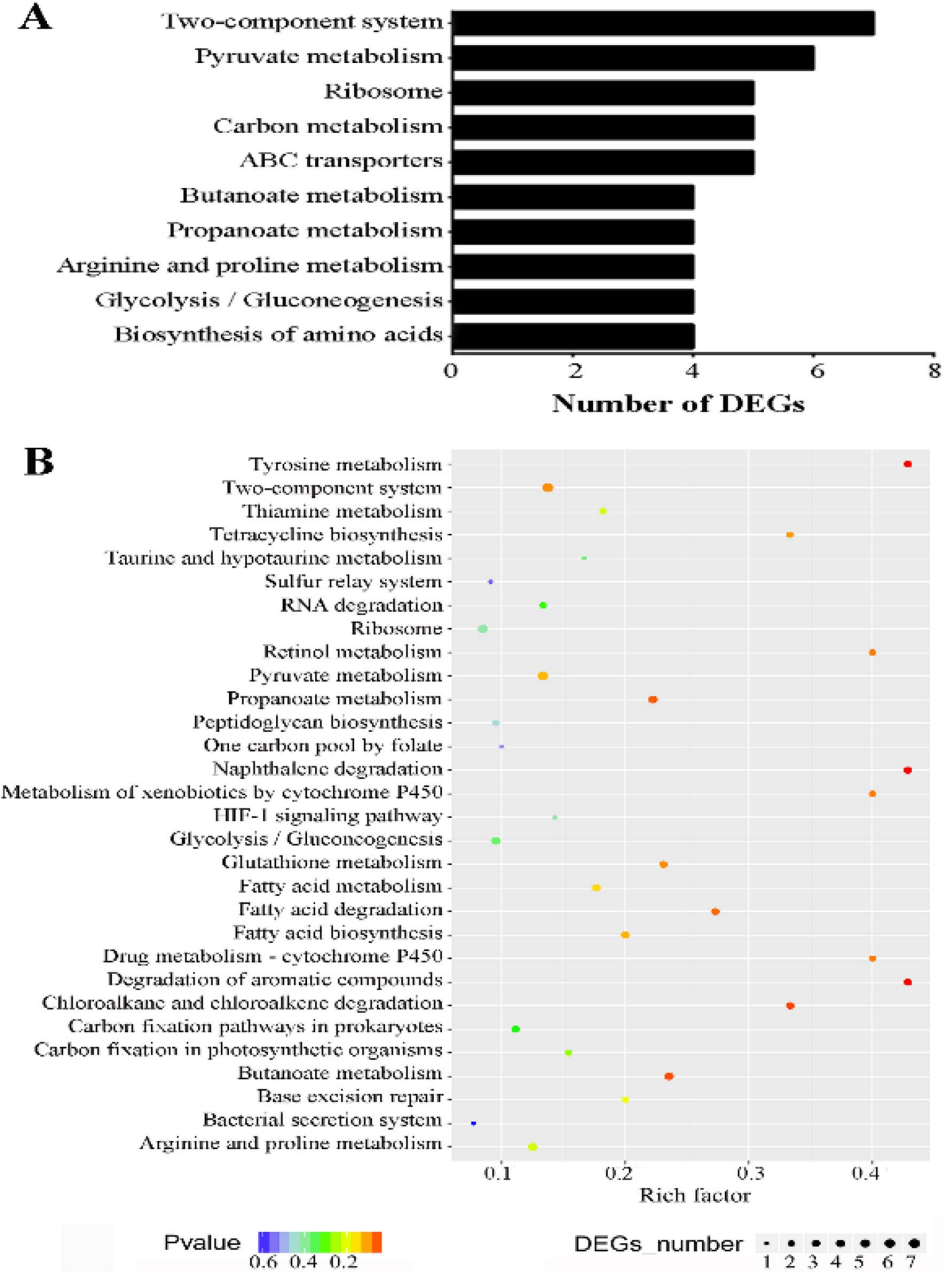
Kyoto Encyclopedia of Genes and Genomes (KEGG) pathway enrichment analysis of 281 differentially expressed genes. (**A**) The top 10 pathways were identified according to the number of enriched genes. The pathway of two-component system had the greatest number of genes, followed by the pyruvate metabolism pathway. (**B**) The top 30 (558) pathways were identified according to the *p* value, rich factor, and the number of enriched genes.

KEGG pathway was used for a biological pathway-based analysis, in which all genes were mapped to the reference canonical pathways of KEGG. The pathways of “degradation of aromatic compounds (Ko01220)”, “naphthalene degradation (Ko00626)”, “tyrosine metabolism (Ko00350)”, ”chloroalkane and chloroalkene degradation (Ko00625)”, and “butanoate metabolism (Ko00650)” were significantly enriched (*p* ≤ 0.05) (Fig. 6B). The pathways of “two-component system (Ko02020)” and “pyruvate metabolism (Ko00620)” were remarkably enriched with more DEGs but without strong significance (*p* > 0.05) (Fig. 6A). These two pathways should not be ignored because of their importance in exploring the pathogenesis and antibiotic resistance of *S. epidermidis*^37,38^. More information about the results of KEGG enrichment is presented in Supplementary Table S3.

## Discussion

Based on the analysis of 16S rDNA, many different types of microorganisms have been found on the ocular surface, mainly including harmless commensal organisms and opportunistic pathogens^39,40^. *S. epidermidis,* formerly considered commensal, has been recognized as an important opportunistic pathogen in nosocomial infection although it is commonly isolated from the skin and mucous membranes of healthy individuals^41,42^. The differences between commensal and nosocomial *S. epidermidis* isolates were reported at the genetic level. A genome sequencing study suggested that commensal skin *S. epidermidis* isolated from different parts of the body of healthy individuals showed a high amount of genetic variation^43^. As for ocular infection isolates, significant genetic variations were detected between 42 isolates of keratitis and endophthalmitis and 14 healthy conjunctival isolates by using fluorescence-amplified fragment length polymorphism^20^. Differences were also found in genotype and phenotype between healthy conjunctival isolates and ocular infectious isolates^19^. In this study, we used RNA-Seq for transcriptome analysis to explore the DEGs of *S. epidermidis* isolated from the healthy conjunctiva and postoperative endophthalmitis. In total, 142 significantly upregulated and 139 significantly downregulated genes were identified in the strains from endophthalmitis. After GO and KEGG functional analyses of these DEGs, we detected several critical genes that may serve key roles in the pathogenesis and therapeutic targets of endophthalmitis caused by *S. epidermidis.*

*S. epidermidis* is generally believed to produce no aggressive toxins. However, aggressive members of PSMs have been identified in *S. epidermidis*^16,17^. PSMs, a family of proinflammatory peptides produced by most staphylococci, have multiple functions in the production of proinflammatory cytokines, immune evasion, biofilm development, cytolytic capacity, and even the killing of competing microbes^44^. Consistently, our results showed that the gene code for phenol-soluble modulins β1 (SE0846) was significantly upregulated in the strains from postoperative endophthalmitis (log_2_FC≈1.49). Meanwhile, another gene SE1634, which codes for Staphylococcal toxin, was also found highly upregulated in the group of P-Se (log_2_FC≈1.76). Staphylococcal toxins possess broadly cytolytic properties and strong proinflammation, which leads to tissue degradation and cell death^15,45^. The expression level of SE1634 was validated by qRT-PCR. Thus, it is inferred that the two genes may be directly responsible for the postoperative endophthalmitis caused by *S. epidermidis*.

At present, qRT-PCR is still considered the method of choice for validation of gene expression data obtained on high-throughput profiling platforms. In this study, ten DEGs were randomly selected to validate the results of RNA-Seq. However, there are minor differences between both experimental conditions. The RNA-Seq processing workflows, the annotation of the reference transcriptome, reads mapping, primer design, and reagents have impacts on the results^46^. Therefore, careful validation is warranted when expression profiles are evaluated with this specific gene set.

GO functional annotation of the transcripts in the current study revealed that both up- and down-regulated genes were related to the metabolic process. The results of enrichment analysis indicated that the terms of homeostatic process, cellular homeostasis, and cell redox homeostasis were significantly enriched in the BP. By analyzing these involved genes, we found that most of the DEGs presented an upregulated trend. In detail, SE2097 and SE0594 codes for thioredoxin and SE0838 code for thiol reductase thioredoxin participated in the thioredoxin system and were significantly upregulated in the group of P-Se (log_2_FC ≈ 1.59, 1.36, and 1.90, respectively). The thioredoxin system, composed of thioredoxin, thioredoxin reductase (TrxR), and nicotinamide adenine dinucleotide phosphate (NADPH), is widely distributed in natural organisms and serves in defense against oxidative stress^35,47^. Moreover, the obvious differences in structure and reaction mechanisms of TrxR between bacteria and mammals have made this system a novel antibiotic target^35^, for example, in *Bacillus anthracis* as a new drug target to several important human pathogens^48^ and in *S. aureus* as a feasible target for antibacterial drug design^49^. Thus, the thioredoxin system may be speculated as an attractive antibiotic target for treatment of endophthalmitis induced by *S. epidermidis*. Furthermore, several genes involved in the iron ion metabolism need to be mentioned. Iron is an essential micronutrient for the growth and proliferation of all organisms, including pathogenic bacteria, and plays a pivotal role in colonization and subsequent pathogenesis^36,50^. *S. aureus* has been confirmed to evolve sophisticated strategies to obtain iron during infection, suggesting the iron acquisition system might be a viable target for therapeutic interventions^36^. In this study, the DEGs SE1764, SE1578, and SE1783, which were involved in the iron ion metabolism, were significantly upregulated in the strains of *S. epidermidis* isolated from postoperative endophthalmitis.

Moreover, the results of KEGG pathway enrichment analysis indicated that pathways of the two-component system and pyruvate metabolism enriched more DEGs. The two-component system contains a membrane-associated histidine kinase and a response regulator to regulate bacterial adaptation, survival, virulence, and biofilm formation^51^. For opportunistic bacterial pathogens, the existence of essential two-component signal transduction systems is a core element of virulence and antibiotic resistance, suggesting that these systems may be targets for antimicrobial interventions^37^. In the present study, seven DEGs were enriched into this pathway, with SE1637 code for histidine kinase and SE1635 code for accessory protein regulator protein B both significantly upregulated in the strains from postoperative endophthalmitis. In addition, data from this study indicated that six DEGs were enriched into the pyruvate metabolism pathway. Pyruvate, a critical metabolite in a variety of anabolic and catabolic pathways including the oxidative metabolism, gluconeogenesis, and tricarboxylic acid cycle, is essential for basic activities of organisms^52^. Harper et al. demonstrated that pyruvate induced robust leucocidin production and enhanced the pathogenicity of *S. aureus*, so it may serve as a novel regulatory signal to coordinate *S. aureus* virulence through intricate regulatory networks^38^. It was also reported that pyruvate was a physiological ligand participating in regulating the network of sporulation in *Bacillus subtilis*^53^. However, the role of pyruvate metabolism in *S. epidermidis* is still not clear.

Our study has several limitations. First, we only collected five *S. epidermidis* isolates from post-cataract endophthalmitis because this type of endophthalmitis has an extremely low incidence, ranging from 0.033 to 0.36%^1–3^, and the positive rate of bacterial culture is also not high. Second, we did not analyze the bacterial phenotype of these isolates in details, which is important for a comprehensive understanding of microorganisms^16,54^. Third, we just explored the differences at the transcriptome level of *S. epidermidis* isolated from different pathological statuses, but did not carry out genome analysis. Fourth, lots of implications of genes are thought to contribute to *S. epidermidis* endophthalmitis, and the corresponding verification needs to be implemented in the future investigations.

In conclusion, the present study identified 281 DEGs of *S. epidermidis* isolates from postoperative endophthalmitis and the healthy conjunctiva using RNA-Seq. With GO and KEGG pathway analyses, several important DEGs and their potential biology pathways were revealed. The findings not only help to understand the pathogenesis of *S. epidermidis,* but also provide a basic resource for identification of therapeutic targets in postoperative endophthalmitis caused by *S. epidermidis*.

## Supplementary information

Supplementary Table S1.

Supplementary Table S2.

Supplementary Table S3.
